# Recent progress and potential future directions to enhance biological nitrogen fixation in faba bean (*Vicia faba* L.)

**DOI:** 10.1002/pei3.10145

**Published:** 2024-05-21

**Authors:** Tamanna Jithesh, Euan K. James, Pietro P. M. Iannetta, Becky Howard, Edward Dickin, James M. Monaghan

**Affiliations:** ^1^ Centre for Crop and Environmental Science Harper Adams University Edgmond, Shropshire UK; ^2^ The James Hutton Institute Dundee UK; ^3^ Universidade Católica Portuguesa, Centro de Biotecnologia e Química Fina (CBFQ), Laboratório Associado, Escola Superior de Biotecnologia Porto Portugal; ^4^ Processors and Growers Research Organisation Peterborough UK

**Keywords:** biological nitrogen fixation, faba bean, legume, mineral nutrition, residual nitrogen, rhizobia, yield variability

## Abstract

The necessity for sustainable agricultural practices has propelled a renewed interest in legumes such as faba bean (*Vicia faba* L.) as agents to help deliver increased diversity to cropped systems and provide an organic source of nitrogen (N). However, the increased cultivation of faba beans has proven recalcitrant worldwide as a result of low yields. So, it is hoped that increased and more stable yields would improve the commercial success of the crop and so the likelihood of cultivation. Enhancing biological N fixation (BNF) in faba beans holds promise not only to enhance and stabilize yields but also to increase residual N available to subsequent cereal crops grown on the same field. In this review, we cover recent progress in enhancing BNF in faba beans. Specifically, rhizobial inoculation and the optimization of fertilizer input and cropping systems have received the greatest attention in the literature. We also suggest directions for future research on the subject. In the short term, modification of crop management practices such as fertilizer and biochar input may offer the benefits of enhanced BNF. In the long term, natural variation in rhizobial strains and faba bean genotypes can be harnessed. Strategies must be optimized on a local scale to realize the greatest benefits. Future research must measure the most useful parameters and consider the economic cost of strategies alongside the advantages of enhanced BNF.

## Graphical abstract

The cultivation of faba beans offers economic and environmental benefits as a result of biological nitrogen fixation (BNF). In this review, we summarize the recent progress in enhancing BNF in the crop and suggest directions for future research.

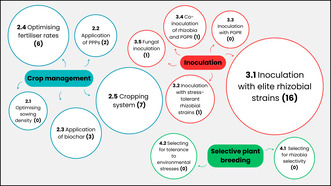

## 1 INTRODUCTION

**FIGURE 1:**
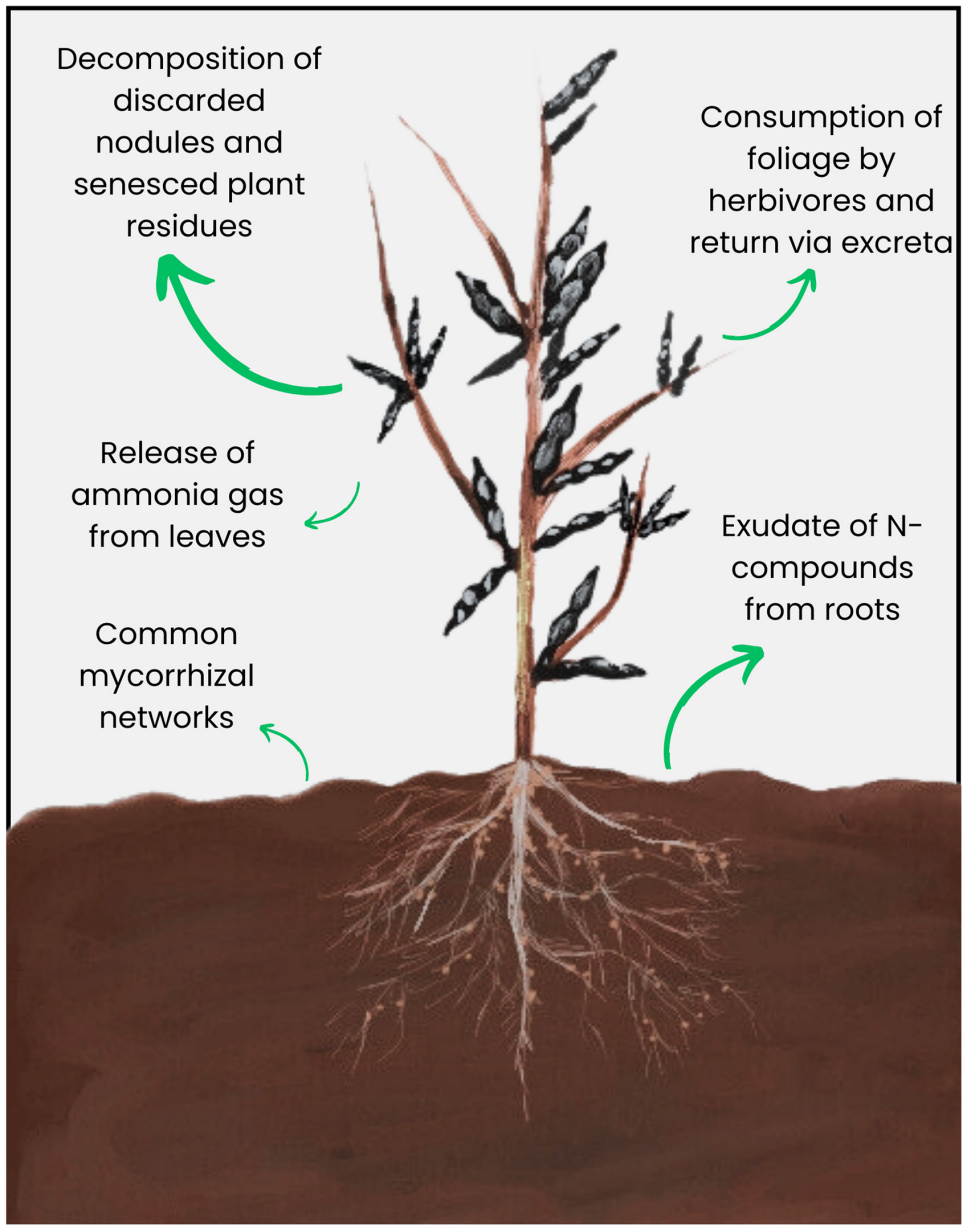
The major routes of nitrogen (N) transfer from the legume to the non‐legumes in cropping systems. The size of the arrow indicates the importance of that route as a means of N transfer, based on Thilakarathna et al. (2016) and Peoples et al. (2015).

Faba beans (*Vicia faba* L.), also known as fava, horse, tic, or field beans, are a globally important grain legume crop. In 2021, 6 M t of faba beans were harvested globally. China is the leading producer, followed by Ethiopia and the UK (FAOSTAT, 2023). An extensive review has covered the various environmental and economic benefits of faba beans in cropping systems (Jensen et al., 2010). Many of these arise as a result of their capacity for obtaining their own nitrogen (N) via the process of biological nitrogen fixation (BNF), which is one of the most ecologically important biochemical reactions on a global scale (Unkovich et al., 2008). BNF in legumes results from a symbiosis they establish with diazotrophic soil bacteria (“rhizobia”) housed within specialized structures mainly on their roots called nodules. Faba bean nodulates most widely with certain strains of *Rhizobium leguminosarum* symbiovar *viciae* (Rlv), which are now considered to be part of the *Rhizobium leguminosarum* complex (Rlc) (Young et al., 2021). The crop exhibits substantial BNF ability; under favorable conditions, faba beans can derive up to 96% of their N from the atmosphere (Palmero et al., 2022). Moreover, they lower the N demand of non‐legume crops in cropping systems through a range of above‐ and below‐ground mechanisms (Figure 1).

Despite these benefits, the global harvested area for faba beans has halved over the past six decades, from 5.4 M ha in 1961 to 2.7 M ha in 2021 (FAOSTAT, 2023). This decline in area sown partly reflects the global shift in farming practices in the latter half of the 20th century from legume rotations as a source of N to crops which are dependent on synthetic N fertilizers. Moreover, as for the majority of legume crops, the low yields of faba beans are considered a major deterrent to farmers to incorporate the crop into their rotations more widely (White et al., 2022). However, it is hoped that higher crop yields will encourage farmers to cultivate faba beans more frequently.

Although numerous factors contribute to yield in faba beans, there is an established correlation between the amount of nitrogen fixed and yield (Maluk et al., 2022). Hence, it is practical to focus on improving BNF as a means of achieving higher and more stable yields for this crop. In addition to lower yields, low BNF may result in legumes such as faba bean contributing negatively, rather than positively, to the soil N balance (Nebiyu, Vandorpe, et al., 2014). Optimizing BNF in legumes, therefore, is proposed as a valuable tool for sustainable agriculture (Del Papa et al., 2024).

Historically, research on BNF has primarily focused on the development of methodologies to determine the quantity of N fixed via BNF per unit area, hereafter referred to as BNF in this review (kg N ha^−1^) (El‐Ghandour & Galal, 1997; Papastylianou, 1987; Senaratne & Hardarson, 1988). A comprehensive and widely scientifically accepted tool for BNF is now widely used, specifically the ^15^N natural abundance technique (Unkovich et al., 2008), so it can be argued that any further studies on a similar vein may no longer be necessary.

Previous studies that specifically examine the response of BNF to changing factors have been largely exploratory. Limited research has examined the effects of crop management practices, such as inoculation with *Rhizobium* or fertilizer input, with varying degrees of success (Smith et al., 2009; Talaat & Abdallah, 2008). Many of the environmental factors and management practices contributing to BNF were described in an extensive review more than a decade ago, in the broader context of cropping systems involving the crop (Jensen et al., 2010). Therefore, given the unexplored opportunity to optimize BNF in the crop (Del Papa et al., 2024), there is a clear need for an updated review of the state of the literature, with clear signposts for directions for future research.

To conduct the literature search, we utilized Google Scholar and Web of Science. The search terms employed to find literature on faba beans were ‘faba bean,’ ‘broad bean,’ ‘field bean,’ ‘vicia faba,’ in combination with ‘nitrogen fixation,’ ‘BNF,’ ‘SNF,’ ‘rhizobi*,’ or ‘nodule’. Additional papers on other legumes were found ad hoc using a similar method if the literature was missing for faba beans. Generally, only papers published in the last decade, starting from February 2024, are reported to capture the most recent state of literature. Given the difficulties and costs associated with measuring BNF (Unkovich et al., 2008), many papers in the literature opt to measure related and correlated parameters such as the number of (effective) nodules or nodule dry weight. Where studies report BNF, values are reported as either the percentage of N in plant tissue derived from the atmosphere (%Ndfa) and/or the amount of N fixed (De Notaris et al., 2023). As the latter is a function of the former and total N yield per unit area, the few studies that report only %Ndfa are omitted from this review since this parameter does not give a useful indication of the quantity of N fixed, which is more important in the context of agriculture and environment as this value quantifies the N services provided by the grain legume.

**FIGURE 2:**
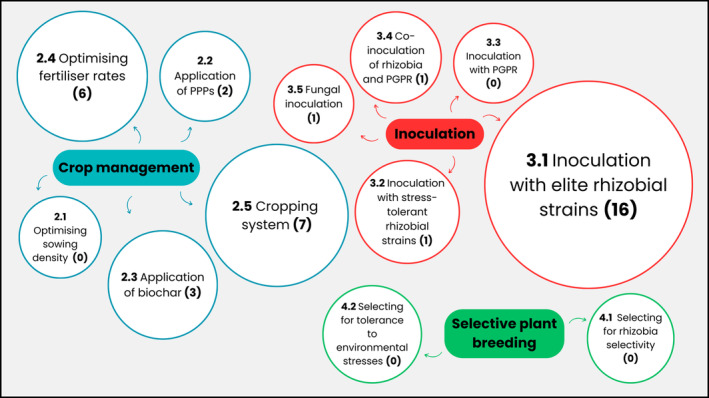
Simplified representation of factors that can be targeted to enhance biological nitrogen fixation (BNF) in faba beans (*Vicia faba* L.). The first number in each circle (e.g., 2.1) relates to the subsection in the review that covers that factor. The size of the circle is related to the number of research publications that have attempted to enhance biological nitrogen fixation (BNF) and/or nodulation by targeting the factor; the number in parentheses in each circle quantifies the number of papers. PPP, plant protection product; PGPR, plant growth‐promoting rhizobacteria.

**FIGURE 3:**
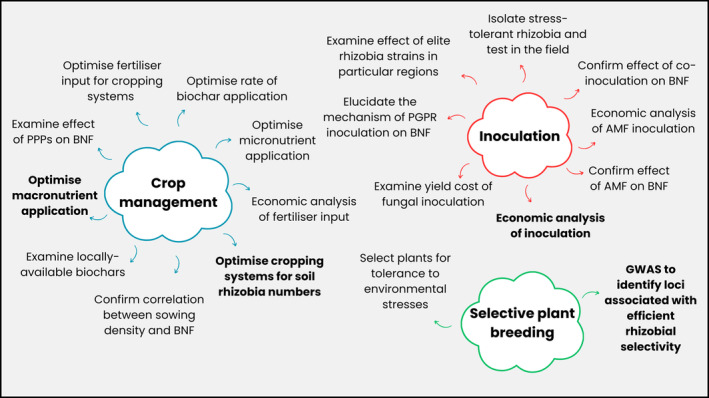
Schematic diagram to show some of the potential directions of future research to increase biological nitrogen fixation (BNF) in faba beans (*Vicia faba* L.). Crop management, inoculation, and plant breeding can be targeted in the long term and the short term to enhance this important parameter. The most important directions for research are given in **bold**.

The aims of this paper are twofold: (1) to assess recent advancements in enhancing BNF in faba beans (Figure 2) and (2) to suggest potential avenues for future research to allow the crop to realize its full global potential (Figure 3).

## 2 CROP MANAGEMENT

### 2.1 Optimizing sowing density

As BNF is a function of N yield per unit of dry plant biomass, optimizing cultivation practices to maximize plant biomass may offer significant benefits to enhancing BNF. One way of achieving this is by optimizing sowing density, which would be relatively simple for growers to implement. Sowing at too low of a density leads to suboptimal yields due to patchy establishment, but sowing at densities that are too high leads to increased competition and a consequent decrease in the number of established plants (Sobko et al., 2019). There have been no recent peer‐reviewed papers that have assessed the effect of sowing density on BNF in faba beans (Figure 2) since Sprent and Bradford (1977) revealed that BNF decreases with higher sowing densities at all the growth stages measured in the small‐seeded faba bean Maris Bead. The effect of sowing density on BNF in other legumes is inconsistent. For example, in soybeans, a study in Central Europe found the highest BNF rates from the highest sowing densities (Radzka et al., 2021), whereas in China, the opposite trend was observed (Wei et al., 2021). This may reflect the variation in optimal sowing density between varieties and growing regions for plant biomass and yield as a result of varying growth habits and/or environmental factors (Gezahegn et al., 2016; Tamrat, 2019). To enhance BNF, therefore, it is necessary to conduct localized studies that focus on optimizing the sowing density of newer varieties of faba beans. These studies should aim to maximize plant biomass but must also measure BNF directly to confirm the positive correlation between sowing density, yield, and BNF in faba beans (Figure 3).

### 2.2 Application of plant protection products (PPPs)

Another crop management practice that can be optimized to enhance BNF is the application of plant protection products (PPPs). The term “PPP” encompasses all chemicals used to control pests, weeds, and diseases including insecticides, fungicides, and herbicides. In addition to their effects on plant growth, the effects of PPPs on BNF may be largely a result of their impact on the survival and efficacy of rhizobia in the soil (Burul et al., 2022). For example, studies based on other crop species have shown that the input of chemicals into agricultural soils can decrease the diversity, abundance, and reproduction of rhizobial species (Johnsen et al., 2001) or increase their reproduction, perhaps by acting as a substrate for their growth (Trimurtulu et al., 2015). Generally, in faba beans, it appears that PPPs have no significant effect. Glyphosate, which is among the world's most widely used herbicides, sprayed in pots 2 days before sowing did not affect nodulation or BNF of faba beans (Aynalem & Assefa, 2017). Similarly, no negative effect was found of the application of the herbicide pendimethalin on the nodulation process (Ntatsi et al., 2018). There is limited literature examining the effect on BNF in the field of most other PPPs approved for use in faba beans (Figure 2). However, other studies report no effect of several commonly used PPPs on the symbiosis or nodulation with related legumes such as peas (*Pisum sativum*) and common beans (*Phaseolus vulgaris*) (Laabas et al., 2022; Oliveira et al., 2016). Given that the use of PPPs is standard agricultural practice for faba bean cultivation in most climates to maximize crop productivity, it may be useful to examine how these affect rhizobial populations and BNF in more detail, despite current evidence suggesting that the use of many of these PPPs may not reduce BNF in this crop (Figure 3). However, this should not be considered a priority for research.

### 2.3 Application of biochar

The potential of biochar in improving BNF in legumes has been covered in detail in a recent meta‐analysis (Farhangi‐Abriz et al., 2022). Generally, increases in BNF and associated nodulation parameters are greater when biochar is added to sandy and loamy soils with low N, carbon (C), and cation exchange capacity (CEC). The addition of the C‐rich soil amendment is thought to contribute positively to the physicochemical properties of the soil, thereby optimizing conditions in the rhizosphere. Indeed, amelioration of soil pH and plant nutrient status was reported to contribute to enhanced BNF in faba beans grown in acidic soils with the addition of biochar (Van Zwieten et al., 2015). Biochar derived from a variety of sources, including soybean straw, maize, papermill, and poultry shed waste, has been shown to increase nutrient uptake of faba beans and therefore contribute to increased nodule number and/or nodule activity (Egamberdieva et al., 2020; Mohamed et al., 2017; Van Zwieten et al., 2015) (Figure 2). Given the growing interest in biochar as a low‐cost soil amendment for agriculture (Das et al., 2023), its routine application in regions with unproductive or infertile land growing faba beans may be beneficial for enhanced BNF. For example, it may be beneficial to determine which locally available source of biochar offers the greatest benefits in terms of BNF or to determine the optimum timings and/or rate of its application to the crop (Figure 3).

### 2.4 Application of fertilizer

When nutrient levels are deficient, nodulation is limited as a result of the important role these nutrients play in the biochemistry of BNF, which is a highly energetically costly process. For example, N‐fixing legumes have high phosphorus (P) requirements as a result of the ATP demand of BNF (Haling et al., 2016). Similarly, reduced BNF under sulfur (S) starvation was found to be due to limitations in ferredoxin, leghemoglobin, and ATP supply (Scherer, 2008). On the other hand, when nutrient levels are high, BNF is also limited. In the case of N, this may be attributed to faba beans not engaging in BNF because of the high energy and carbon costs associated with the symbiosis compared to the uptake of N from the soil as nitrates or ammonium (Mohammadi et al., 2012). Nevertheless, compared to other legumes, faba beans are observed to maintain BNF at high soil N levels (Guinet et al., 2018; Rose et al., 2016) providing the rate of photosynthesis is sufficient (Etemadi et al., 2019).

A summary of recent studies attempting to optimize macronutrient fertilizer input for nodulation and BNF in faba beans is given in Table 1. Among the macronutrients, the optimization of N, P, and potassium (K) has received substantial attention in the literature. The pattern appears to be that optimum recommendations for fertilizer rates must return levels of nutrients in the soil to a particular optimum level. For example, in studies that have focused solely on optimizing P in the field, optimum rates vary from 20 kg P ha^−1^ (Amanuel et al., 2000) to 40 kg P ha^−1^ (Desta et al., 2015) for enhancing nodule numbers in Ethiopia, where these were the highest rates tested in the respective experiments. This difference could be explained by the initial difference in the availability of P; the soil where 40 kg P ha^−1^ was optimum had a third of the P present before fertilization compared to soils with a higher P baseline where 20 kg P ha^−1^ was found to be optimum. The optimum P application rates, therefore, varied according to the amounts already present in the soil, as well as other environmental factors specific to growing regions, within the same country.

**TABLE 1:** A summary of recent studies optimizing macronutrient fertilizer input for nodulation and biological nitrogen fixation (BNF) in faba beans (*Vicia faba* L.) and the associated effect on crop yield.

Reference	Country of study	Nutrient(s) studied	Treatments (kg ha^−1^)	Optimal treatment (kg ha^−1^)	Effect on nodulation compared to control	Effect on BNF compared to control	Effect on yield compared to control
Desta et al. (2015)	Ethiopia	P	P: 0, 20, 40	Not specified	Increase in nodule number, nodule dry weight, and nodule volume with all rates	Not assessed	Increased with all rates
Mesfin et al. (2020)	Ethiopia	N, P	N: 0, 10, 20, 46 P: 0, 10, 20	N: 20, P: 20	Increase in nodule number by 129% and nodule dry weight by 425%	Increase in effective (pink) nodules by 35% and BNF (kg N ha^−1^) by 67%	Increased by 46%
Adak and Kibritci (2016)	Turkey	N, P	N: 0, 30, 60, 90 P: 0, 40, 80	P: 40 + N: 60	Decrease in nodule number by 7% and nodule weight by 31%	Not assessed	Increased by 99%
Hanoon et al. (2020)	Iraq	N	N: 62.5, 125, 250	Not specified	Increase in nodule number and nodule dry weight with all rates	Not assessed	Increased with all rates
Pampana et al. (2018)	Italy	N	N: 0, 40, 80, 120, 160	N: 0	Decrease in nodule dry weight by 43%	Decrease in BNF (g N m^−2^) by 27%	No significant change at any rate
Niewiadomska et al. (2015)	Poland	K, S	K: 0, 40, 80, 160 S: 0, 25, 50	K: 25 + S: 25	Not assessed	Increase in nitrogenase activity by 168%	Not assessed

Abbreviations: K, potassium; N, nitrogen; P, phosphorus; S, sulfur.

This is also true for fertilizers applied in combination. For example, in Turkey, Adak and Kibritci (2016) reported that a combination of 80 kg P ha^−1^ and 30 kg N ha^−1^ was optimal for nodulation parameters, while in Ethiopia it was a combination of 20 kg P ha^−1^ and 20 kg N ha^−1^ that was optimum for BNF and nodulation (Mesfin et al., 2020). Both studies report similar levels of P in the soil before sowing. Therefore, the optimal level in the soil is not only determined by the nutrient levels in the soil alone, but also according to other environmental factors. This suggests that fertilizer rates must be optimized on a local scale if they are to ultimately increase BNF (Figure 3).

The direct effect of magnesium (Mg) on BNF in faba beans has not been studied, despite its application being standard agricultural practice in many countries including the UK (AHDB, 2022). Similarly, little attention has been paid to S nutrition of legumes; it is generally not applied to faba beans as standard agricultural practice (AHDB, 2022; GRDC, 2017). Similarly, although yield benefits and increased nodule numbers have been reported following application of boron (B) fertilizer in combination with zinc (Zn) and molybdenum (Mo) (Adissie et al., 2020; Mohamad & Mohammed, 2020), there have been no attempts to optimize the rates of these micronutrients for BNF. Therefore, in addition to optimizing N, P, and K to local regions, future studies focusing on elucidating the roles of Mg, S, and macronutrients such as B and Mo in BNF and optimizing the application of these nutrients may also be beneficial. Any studies optimizing N as “starter N” must also bear in mind the environmental and economic costs associated with its application. In addition, it is also important to conduct an economic analysis of field trials to determine the marginal rate of return of fertilizer inputs, considering both yield and the associated N benefits to subsequent crops (Figure 3).

### 2.5 Cropping system

An alternative approach is to optimize the cropping system for BNF (De Notaris et al., 2023). Given the environmental benefits of sustainable cropping systems, optimizing BNF provides synergistic benefits. There is increased interest globally in no‐tillage and organic farming systems reflecting the shift toward more sustainable agricultural practices (Singh et al., 2023; Yue et al., 2023). These practices may be beneficial for BNF in faba beans but not always (De Notaris et al., 2023), perhaps suggesting the importance of optimal seasonal growing conditions too. For example, cultivating faba beans under two Mediterranean no‐tillage systems increased BNF (Simon‐Miquel et al., 2024; Tedone et al., 2023). Similarly, the addition of sheep manure (Yfantopoulos et al., 2022) and municipal compost (Maluk et al., 2022) is both reported to lead to increased BNF compared to the conventional mineral synthetic PK inputs in Greece and Scotland, respectively. These findings can be attributed to these farming systems creating more favorable conditions in the soil for rhizobial growth and colonization (Tedone et al., 2023) as well as lower levels of soil N (Yfantopoulos et al., 2022). Future studies, therefore, may consider quantifying complementary soil rhizobia or soil N under various cropping systems as a means to determine BNF potential in a particular field (Figure 3).

Unlike the no‐tillage cropping system, legume/cereal intercropping is an increasingly prevalent sustainable cropping practice that may negatively affect BNF in faba beans in some circumstances (Rodriguez et al., 2020); their meta‐analysis found that although the %Ndfa in faba beans increased in intercropped systems, BNF may decrease under certain conditions, depending on additional factors such as the input of N fertilizer. This may be attributed to the lower biomass of the legume crop in intercropped systems compared to a sole crop of legume. Compared to other N‐fixing legumes, BNF in faba beans in intercropped systems is less susceptible to inhibition after the application of synthetic or mineral N fertilizer (Guinet et al., 2018; Rose et al., 2016). Nevertheless, BNF was inhibited in intercropping systems where mineral N was applied at rates of over 100 kg N ha^−1^ (Fan et al., 2006; Rose et al., 2016). However, Mei et al. (2021) reported increased BNF in faba beans intercropped with maize with rhizobial inoculation and the addition of synthetic N at 150 kg N ha^−1^ in reclaimed desert soil in China. Therefore, BNF benefits due to intercropping may only be realized in particular growing regions and conditions. Optimizing fertilizer input and sowing rates for biomass of faba beans in intercropping systems involving the crop will mutually benefit the partner cereal crop as a result of enhanced BNF leading to greater N transfer (Figures 1, 3).

## 3 INOCULATION

### 3.1 Inoculation with elite rhizobial strains

Given the variation in rhizobial compatibility with legumes, and therefore symbiotic effectiveness, the maximum BNF capacity of faba beans, and legumes in general, is rarely reached in most agricultural settings (Allito et al., 2020; Mekonnen & Mnalku, 2021). It is therefore suggested that “elite” Rlv strains (i.e., strains that have the potential to increase legume growth and BNF above than allowed by other strains), applied as inoculants, might be favored by the host legume over strains that are less efficient in the symbiosis in the soil, leading to increased BNF (Maluk et al., 2022; Westhoek et al., 2021). Therefore, in addition to sufficient numbers of complementary soil rhizobia, it is also important that the complementary strains of rhizobia are present for optimal BNF.

In the UK, it was observed that appropriate strains of Rlv persist in agricultural soils for decades, even in the absence of legume cropping (Maluk et al., 2022), and this may be true for other temperate regions, especially those in which relatives of faba beans (other *Vicia* spp. and *Lathyrus* spp.) are native, possibly negating the need for inoculation. Indeed, increases in nodulation, BNF, and yield as a result of inoculation are not always found (Fogelberg et al., 2023; Maluk et al., 2022; PGRO, 2023), and hence, inoculation is not recommended as standard practice, especially in Europe. Interestingly, effective inoculation can be limited, even in regions where *Vicia* spp. are not native and in which inoculation is generally recommended, such as in Australia. This is thought to result from the presence of less effective rhizobia already present in the soil from previous inoculations, since Denton et al. (2013) only showed increased BNF when the inoculant was applied at 100 times the normal rate.

The presence of appropriate complementary rhizobial strains in the soil remains an important determinant of BNF in faba beans and other legumes such as soybean (Maluk et al., 2023). Although inoculation is yet to show increased BNF and/or a yield benefit in European studies, numerous field studies elsewhere, particularly in Africa, report significantly increased nodulation and/or BNF when inoculating soil or faba bean seeds with certain Rlv strains (Table 2). Considering these factors, future research on inoculation to increase BNF should also consider the economic cost of the practice and should be a priority in regions where complementary (i.e., potential competitor) strains are not native to the soil (Figure 3).

### 3.2 Inoculation with stress‐tolerant rhizobial strains

The physiological mechanisms of the adverse effects of abiotic stresses such as drought, salinity, and heat on BNF in legumes have been summarized recently (El Sabagh et al., 2020). Extended periods of drought cause a decrease in soil *Rhizobium* populations, which is often associated with poor nodulation of legumes during dry seasons (Atieno & Lesueur, 2019; Mohammadi et al., 2012). This further reinforces the recurring theme that quantification of soil rhizobia is a useful determinant of BNF potential (Figure 3). Decreased BNF can also in part be attributed to hypoxia as a result of compaction of the nodule structure during drought stress, leading to perturbed respiration (Chammakhi et al., 2022).

Inoculation may offer benefits in faba bean growing regions that frequently experience environmental stresses such as temperature extremes, desiccation, drought, salinity, pH, and heavy metals. The isolation of Rlv strains that survive in soil and can nodulate under stress and their subsequent assessment in the field under abiotic stress may be a useful future avenue for research. For example, L'taief et al. (2019) found that inoculation with salt‐tolerant *Rlv* strains increased nodulation and shoot N in faba beans in the presence of salinity in a glasshouse study, presumably as a result of enhanced rhizobial survival. Moreover, given that both the plant and the bacteria are important in the symbiotic relationship, potentially combining a tolerant faba bean genotype with an appropriate tolerant Rlv strain may provide the greatest opportunity to enhance BNF under a particular environmental stress (Figure 3). These stresses will become more prevalent due to global climate change (Beacham et al., 2018), making it increasingly important to harness the natural variation in both faba bean and rhizobial populations to safeguard the future production of the crop.

### 3.3 Inoculation with plant growth‐promoting rhizobacteria (PGPR)

PGPR act in the rhizosphere surrounding roots and benefit plant growth in a variety of direct and indirect ways (Mohanty et al., 2021). The exact mechanism by which BNF is enhanced by PGPR inoculation is poorly understood, but the suppression of pathogens and solubilization of phosphate, both of which enhance BNF, may play a role. Indeed, in faba beans, inoculation with PGPR has been shown to increase resistance to diseases (Abdelkhalek et al., 2022) and tolerance to salinity stress (Metwali et al., 2020), which may indirectly enhance BNF, although this parameter was not directly measured in the mentioned studies. Presently, no studies that the authors are aware of have assessed the effect of PGPR inoculation on BNF in faba beans (Figure 2), although work in other legumes suggests a potential benefit. For example, in the common bean (*Phaseolus vulgaris* L.), nodule numbers, nodule dry weight, and, most importantly, BNF were increased as a result of inoculation with PGPR strains (Yadegari et al., 2010). Future studies, therefore, need to gain a deeper understanding of the mechanism by which PGPR inoculation enhances BNF. This requires BNF and associated parameters to be measured directly in studies involving PGPR inoculation of faba beans, in addition to the measurement of the effect of PGPR activity such as disease resistance and tolerance to stresses. Understanding the mechanism will enable a more focused approach to selecting the PGPR strains with the most beneficial properties for enhancement of BNF to further test on faba beans (Figure 3).

### 3.4 Co‐inoculation of rhizobia and PGPR

It is thought that when applied together, PGPR strains increase root growth of the legume to allow more sites for nodulation by rhizobia, therefore leading to a synergistic increase in BNF (Barbosa et al., 2021). Indeed, in soybean cropping in South America, the co‐inoculation of its nodulating symbiont *Bradyrhizobium* with the well‐studied PGPR *Azospirillum brasilense* is now standard practice, as it not only improves BNF and grain yield, but also confers greater crop tolerance to water stress (Prando et al., 2024). However, this is not yet the case with other crop legumes, such as faba beans, even though there is increasing evidence for its potential benefits. For example, co‐inoculation with one Rlv and two PGPR strains led to significant increases in nodule number, plant growth, and shoot N content of faba beans in a pot experiment (Mowafy et al., 2022). The latter finding may suggest increased BNF as a result of the co‐inoculation, although this trait was not specifically measured. In addition, co‐inoculation with a PGPR (*Pseudomonas putida*) and an Rlv strain led to enhanced tolerance to drought and increased yields, although BNF itself was not measured (Mansour et al., 2021). This perhaps suggests that co‐inoculation of PGPR with Rlv strains may indirectly enhance BNF by ameliorating environmental stresses and, therefore, may benefit certain regions globally where these stresses are limiting faba bean production (Kaschuk et al., 2022). As with inoculation with Rlv alone, the economic cost of co‐inoculation must be considered alongside any increases in BNF and/or yield in future studies; that being said, in the case of soybean the costs per dose of the co‐inoculants are sufficiently low that the yield benefits outweigh them (Barbosa et al., 2021; Prando et al., 2024). Moreover, studies must directly measure BNF in order to confirm the benefit of co‐inoculation to this parameter under various conditions (Figure 3).

### 3.5 Fungal inoculation

Inoculation with arbuscular mycorrhizal fungi (AMF) increases P uptake by roots and therefore theoretically enables legumes to meet the high P demand of BNF (Beslemes et al., 2022; Shi et al., 2021). Inoculation with another fungus, a species of white‐rot fungus (*Ceriporia lacerate*), has been shown to increase BNF in faba beans in a pot trial (Yin et al., 2022). Like AMF inoculation, this increase can be attributed to the fungus increasing the availability of nutrients including P in the soil and by inducing changes in root morphology providing more sites for nodule formation. This practice, therefore, may be particularly beneficial in soils with moderate or low levels of P as an alternative to applying additional P fertilizer to enhance BNF. Inoculation of AMF both with and without rhizobia led to increased productivity of faba beans, potentially through increased BNF, although this was not specifically measured (Pereira et al., 2019). The incorporation of these fungal inoculants in crop management practices has the potential to be explored further, assuming similar results are found in field trials. The benefit to BNF as a result of fungal inoculation, however, must first be confirmed. In addition, given the C cost to the plant of associating with both the fungi and the Rlv, the effect of an increased tripartite symbiosis on BNF and yield in the field must be assessed (Figure 3).

## 4 SELECTIVE PLANT BREEDING

Currently, the focus in faba bean breeding for enhanced BNF is generally on selecting varieties that have a high BNF or %Ndfa. As discussed, the latter parameter does not indicate the quantity of fixed N. Moreover, there does not appear to be a clear indication of genetic diversity for %Ndfa among faba bean varieties (Boots‐Haupt et al., 2022), although this could be as a result of limitations of the method of measurement (Nebiyu, Huygens, et al., 2014). The agronomically important parameter, BNF, is additionally influenced by numerous environmental and management factors, which makes it a poor target in breeding programs. For example, although BNF is reported to vary significantly among germplasm (Nebiyu, Huygens, et al., 2014; Neugschwandtner et al., 2021), this trend is not consistent across the literature (Maluk et al., 2022). These findings reflect the fact that other environmental factors associated with the cropping regions are contributing to BNF. Indeed, in the common bean (*Phaseolus vulgaris* L.), a related legume, the heritability of traits associated with BNF varies up to sixfold depending on the stresses present in the environment (Farid et al., 2017).

### 4.1 Selecting for rhizobial selectivity

An alternative, and more effective, way to improve BNF through breeding may be to focus on selecting for faba bean germplasm that effectively selects for the most efficient rhizobial strains in soil with which to engage in symbiosis (Dwivedi et al., 2015; Skovbjerg et al., 2023). The natural diversity of rhizobial strains in agricultural soils is large, and rhizobial strains are not equal in their effectiveness within the symbiotic association with the host plant and, therefore, their BNF capacity (Allito et al., 2020; Maluk et al., 2022). The capacity to select for more efficient strains for nodulation and BNF clearly exists in pea (Westhoek et al., 2021), so it is also likely to occur in its close relative, faba bean. Indeed, there is unexplored variation among faba bean germplasm for strain selectivity (Adhikari et al., 2021), and this strain selectivity may be less influenced by the environment and is, therefore, a potential target for breeding programs (Dwivedi et al., 2015). On this basis, the selection for faba bean germplasms that effectively select for efficient N‐fixing rhizobial strains remains an objective in faba bean breeding programs such as ProFaba (Adhikari et al., 2021).

Although there has not been any progress reported to date (Figure 2), the recent publication of the extensive 13 Gb faba bean genome offers exciting prospects for this genomics‐based breeding platform, poised to accelerate breeding aims (Jayakodi et al., 2023). Moreover, a genome‐wide association study (GWAS) in MAGIC populations has been used to identify genomic regions associated with numerous traits including disease resistance and flowering time (Skovbjerg et al., 2023), raising the possibility of a similar study focusing on loci associated with efficient rhizobial selectivity, which is necessary (Figure 3). Indeed, this approach has already been demonstrated in other grain legumes with promising results in terms of enhanced BNF (Dwivedi et al., 2015).

### 4.2 Selecting for tolerance to environmental stresses

For effective BNF, the growth of both the legume partner and the rhizobial partner in the nodulation symbiosis must be adequate. Environmental stress can cause suboptimal BNF by inhibiting proper growth of the legume.

Like all crops, faba bean varieties vary in their tolerance to different levels of environmental stresses (Mansour et al., 2021). This suggests that there is potential to select for these traits in breeding programs. For example, a faba bean genotype with tolerance to salt in Mediterranean‐type climates has been identified from pot studies (Benmoussa et al., 2022), although it is not clear whether the increased tolerance is a result of increased BNF. Selecting for further genotypes with increased tolerance to particular stresses and subsequently assessing their performance for BNF and yield under field conditions in future studies are a potential route to enhancing these traits in faba beans (Figure 3).

## 5 CONCLUSIONS AND OUTLOOK

The renewed interest in legumes globally is a result of an ever‐pressing need for more sustainable cropping systems. This is shedding light on the importance of enhancing BNF in faba beans, both as a means to potentially stabilize yields and to further reduce the N fertilizer needed for subsequent crops. Most recent progress has been due to optimization of fertilizer input (Section 2.4 and Table 1), rhizobial inoculation with elite strains (Section 3.1 and Table 2), and optimization of cropping systems (Section 2.5).

**TABLE 2:** A summary of studies optimizing inoculant rhizobial strain for faba beans (*Vicia faba* L.) and the associated effect of optimal strains on nodulation, BNF, and yield.

Reference	Type of trial	Country of study	Effect on nodulation compared to uninoculated	Effect on BNF compared to uninoculated	Effect on yield compared to uninoculated
Bhomik et al. (2022)	Field	Bangladesh	Increased nodule dry weight by 28% and nodules per plant by 63%	Not assessed	Increased by 15%
Othman and Tamimi (2016)	Pot	Jordan	Increase in number of nodules by 90% and nodule fresh weight by 500%	Not assessed	Not assessed
Hanoon et al. (2020)	Field	Iraq	Increase in number of nodules by 438% and dry weight of nodules by 636%	Not assessed	Increased by 25%
Mohamad and Mohammed (2020)	Field	Egypt	Increase in nodule fresh weight by 14% and nodules per plant by 27%	Not assessed	Increased by 12%
El Sayed et al. (2015)	Field	Egypt	Increase in nodule number per plant by up to 800%, nodule dry weight by up to 3000%, and average weight of nodule by up to 7800%	Not assessed	Increased by up to 136%
Youseif et al. (2017)	Field	Egypt	Increase in nodule dry weight by up to 4184%	Not assessed	Increased by up to 230%
Woldekiros et al. (2018)	Pot	Ethiopia	Increase in nodules per plant by 66% and nodule dry weight by 52%	Not assessed	Increased by 111%
Adissie et al. (2020)	Field	Ethiopia	Increase in nodules per plant by 41%, nodule volume per plant by 79%, and nodule dry weight by 63%	Not assessed	Increased by 46%
Argaw and Mnalku (2017)	Field	Ethiopia	Increase in nodule number by 71% and nodule dry weight by 184% in one year, no significant difference in another	Not assessed	Increased by 51% (in one year)
Kebede and Lele (2022)	Field	Ethiopia	Increase in nodule number by up to 97%	Not assessed	Increased by up to 137%
Desta et al. (2015)	Field	Ethiopia	Increase in nodule number by 39% and nodule dry weight by 74%	Not assessed	Increased by 38%
Allito, Ewusi‐Mensah, and Logah (2020)	Field	Ethiopia	Increased in nodule dry weight in all faba bean varieties	Increased by up to 177% in BNF	Not assessed
Genetu et al. (2021)	Field	Ethiopia	Increase in total number of nodules by up to 126%	Increase in active (pink) nodules by up to 370%	Increased by up to 80%
Fekadu et al. (2018)	Field	Ethiopia	No significant increase in nodule dry weight or nodule number	Not assessed	Increased by 20%
Geleta and Bekele (2022)	Field	Ethiopia	Not assessed	No significant increase in active (pink) nodules	No significant increase

Abbreviation: BNF, biological nitrogen fixation.

A summary of some of the potential directions for future research to enhance BNF is given in Figure 3. Modification of crop management practices, such as optimization of fertilizer inputs and/or cropping system type, to increase plant biomass and/or BNF will provide relatively easy‐to‐implement solutions. In the long term, a genomics‐based approach to select for faba bean genotypes with greater rhizobial selectivity may be part of the solution. Regardless of the strategy, future research must focus on making the appropriate measurements that can track progress toward the goal, that is, BNF rather than simply %Ndfa. Another recurring theme is not just the presence of complementary rhizobia in the soil, but also their presence in sufficient numbers for adequate BNF. Thus, quantification of soil rhizobia with qPCR or a similar method will be an important parameter to measure in future field studies. Moreover, future studies must also consider the economic cost to growers of implementing particular strategies alongside their environmental and economic benefits. There is evidence, particularly in the case of optimizing fertilizer rates or rhizobial inoculation, to suggest that the optimum strategy for one region may not be the case for another. Therefore, strategies must be optimized on a regional scale for local conditions, rather than blanket recommendations.

Improving BNF in faba bean can have significant economic and environmental benefits, promoting the wider adoption of faba bean agriculture and food and feed systems more broadly, through increased yield potential and decreased yield variability. Success in this regard would also facilitate broader adoption of this underutilized species as a pivotal crop diversification measure via its high potential to also help realize more sustainable and resilient cropped systems regionally and globally.

## FUNDING INFORMATION

TJ was funded by the Biotechnology and Biological Sciences Research Council (BBSRC) as part of the Collaborative Training Program for Sustainable Agricultural Innovation (CTP‐SAI) (Grant BB/W009439/1), in partnership with the Processors and Growers Research Organisation (PGRO). PPMI and EKJ are supported by the Rural and Environment Science and Analytical Services (RESAS), a division of the Scottish Government, the European Commission Research and Innovation Actions www.RADIANT‐project.eu (Horizon 2020, Grant Agreement Number: 101000622), www.econutri‐project.eu (Horizon Europe, Grant Agreement Number: 101081858), and www.legumES‐project.eu (Horizon Europe, Grant Agreement Numbers: 101081858 and 101135512, respectively).

## CONFLICT OF INTEREST STATEMENT

The authors confirmed that there is no conflict of interest to declare.

## Data Availability

Data sharing does not apply as no new data were created or analysed in this review.
